# The Rodent Models of Dyskinesia and Their Behavioral Assessment

**DOI:** 10.3389/fneur.2019.01016

**Published:** 2019-10-11

**Authors:** Qiwei Peng, Shaoping Zhong, Yang Tan, WeiQi Zeng, Ji Wang, Chi Cheng, Xiaoman Yang, Yi Wu, Xuebing Cao, Yan Xu

**Affiliations:** Department of Neurology, Tongji Medical College, Union Hospital, Huazhong University of Science and Technology, Wuhan, China

**Keywords:** dyskinesia, disease models, behavior rating scale, Parkinson's disease, Levodopa, abnormal involuntary movements, rodent

## Abstract

Dyskinesia, a major motor complication resulting from dopamine replacement treatment, manifests as involuntary hyperkinetic or dystonic movements. This condition poses a challenge to the treatment of Parkinson's disease. So far, several behavioral models based on rodent with dyskinesia have been established. These models have provided an important platform for evaluating the curative effect of drugs at the preclinical research level over the past two decades. However, there are differences in the modeling and behavioral testing procedures among various laboratories that adversely affect the rat and mouse models as credible experimental tools in this field. This article systematically reviews the history, the pros and cons, and the controversies surrounding rodent models of dyskinesia as well as their behavioral assessment protocols. A summary of factors that influence the behavioral assessment in the rodent dyskinesia models is also presented, including the degree of dopamine denervation, stereotaxic lesion sites, drug regimen, monitoring styles, priming effect, and individual and strain differences. Besides, recent breakthroughs like the genetic mouse models and the bilateral intoxication models for dyskinesia are also discussed.

## Introduction

Dopamine (DA) replacement therapy with levodopa (L-DOPA) is the most effective pharmacotherapy for motor symptoms of Parkinson's disease (PD). However, prolonged L-DOPA use inevitably leads to complications such as motor fluctuations (i.e., on–off fluctuations and wearing-off phenomenon) and dyskinesia. As the earliest and the most common complication, L-DOPA-induced dyskinesia (LID) occurs in half of the patients undergoing treatment for 5 years (1, 2). Dyskinesia is defined as abnormal involuntary movements characterized by hyperkinetic movements or dystonic features (3). This occurs mostly at the maximal L-DOPA plasma level (peak-dose dyskinesia) and less commonly at the initial or the late phase of drug use, or both (diphasic dyskinesia) (4). It should be noted that dyskinesia can also be induced by other DA agonists or dopaminergic neuron transplant (*viz*., graft-induced dyskinesia, GID) (5), but LID is the most classical type. Although much has been learnt about the risk factors of dyskinesia (i.e., the onset age of PD, disease severity, L-DOPA dosage, and pulsatile administration) (1), its exact molecular mechanisms remain unresolved and there is no effective treatment for PD (6, 7).

Animal model plays an important role in therapeutics research. The 1-methyl-4-phenyl-1,2,3,6-tetrahydropyridine (MPTP)-lesioned non-human primate was the first model of LID. It provides an accurate replication of motor features of human dyskinesia, but the use of this model is largely limited by adverse factors like non-unified methodological criteria and high cost (8, 9). Given the advantages of time- and cost-effectiveness and the ease of genetic manipulation, several rodent dyskinesia models have been developed since the 1990s. However, only few dyskinesia assessment protocols have been validated based on available clinical agents known to have efficacy on LID. In this paper, we discuss the recent advances in modeling dyskinesia in rodents (mainly in unilateral 6-OHDA-lesioned rodents) and analyze the pros and cons of different dyskinesia assessment protocols. We further discuss the major disputes and factors influencing the assessments procedures and the recent advances in the methods used to establish rodent dyskinesia models. This review aims to provide an application framework of the past and present rodent models of dyskinesia to help researchers using the available animal models and behavioral assessment protocols to obtain reliable findings.

## The Development of Rodent Models of Dyskinesia

### 6-OHDA Rat Model

The unilateral 6-OHDA-lesioned rat model is a classical model for PD motor symptoms that was first introduced in the 1960s, soon after which drug-induced involuntary rotation was described in this model (10, 11). Due to the lack of typical motor impairments observed in human parkinsonism, i.e., bradykinesia, rigidity, and rest tremor, rotation has long become the only behavioral output in unilaterally 6-OHDA-lesioned rats and is used to test parkinsonian disability and modeling the response to DA replacement therapy (12, 13).

In the 1970s, Creese and Iversen observed a series of stereotypy responses induced by amphetamine in DA denervated rats and established a rough rating scale where the numbers stand for the presence or absence of a certain response state, i.e., sniffing, licking, and gnawing (14). Since then, the term stereotypy was used to refer to the dopaminergic drug-induced abnormal response in parkinsonian rats (15, 16). However, stereotypy did not fit specific symptomatic analogs in human patients, and its dyskinetic predictive value has been doubted by some scholars (17) as stereotyped behavior may also be induced among normal rats by over-stimulation of the DA system (18).

In the 1990s, scholars found that changes in rotation behavior over time may be a useful model for motor fluctuations (19). Before long, Henry et al. introduced a rotation sensitization model for measuring dyskinesia in rats (20). Actually, response sensitization, *viz*., a gradual increase in rotation or stereotypy response to repeated administration of DA agonists, has been reported in unilateral 6-OHDA-lesioned rats earlier before the 1980s (21, 22), but it was Henry who first linked this abnormal response with dyskinesia since LID also shares a gradual increasing severity over time in the non-human primate models. Although disputed, the theory has been modified and used to investigate the pharmacological efficacy of antidyskinetic agents by some laboratories (23, 24).

Elsewhere, Cenci and colleagues first observed and defined the abnormal involuntary movements (AIMs) in rats after a 2- to 3-week administration of L-DOPA. The features matched with the dyskinetic manifestations of PD patients and non-human primate models (25). Rat AIMs were classified into four subtypes according to the body parts involved, i.e., rotational locomotion, axial torsion, limb movements, and orolingual stereotypies, and each subtype was scored 0–4 separately in accordance with the corresponding time/monitoring period during which the body part was affected (25). However, rotational locomotion differs from the other three subtypes (collectively called ALO AIMs or Body AIMs) due to its unique properties, which are discussed in detail under the subsection “Controversies around the assessment of rodent dyskinesia.” Later on, some modifications were performed on this scale, e.g., addition of amplitude scores for limb and axial AIMs to increase scoring accuracy (26), but such amplitude scores are too complicated to be applied in majority of studies. Cenci's rating scale has since been well-accepted in dyskinesia studies since it was validated by high-quality pharmacological trials and LID molecular markers' tests (25, 27–29). Although detailed rating rules, e.g., the turns and intervals of rating, were constantly revised by subsequent groups (30–32), the framework of this scale remains unchanged.

Similarly, Steece-Collier et al. developed an independent rating scale for rat dyskinesia (33, 34). This scale had more subcategories of dyskinetic movements, i.e., neck postural dysfunction, trunk dystonia, forelimb dystonia, hindlimb dystonia, contralateral forepaw dyskinesia, orolingual stereotypy, and forelimb–facial stereotypy. During the rating session, scores of each item are graded separately based on the intensity and frequency and then multiplied, named as severity scores. The total LID score is calculated by the severity scores. Steece-Collier's scale is mainly used for evaluating LID in mesencephalic dopaminergic neuron graft rats (34–36) and also for GID rating (37).

Thus, far, Henry's, Cenci's, and Steece-Collier's protocols have been used for the behavioral assessment of dyskinesia in rats by other researchers with modifications (24, 30, 38, 39). Being the first model to quantitatively record LID in rats, Henry's protocol differs from the other two in many aspects, and its efficiency is controversial as it is not clear whether rotational behavior is a manifestation of rodent dyskinesia (40). Despite similarities in the rating rules, there are remarkable differences in the rating items and monitoring styles between Cenci's and Steece-Collier's protocols (Table 1). Whereas, Cenci's scale is designed to avoid measuring stereotyped behaviors (orolingual AIM excepted), Steece-Collier's scale is a hybrid of both dyskinetic and stereotypy-like movements scoring (41). The fact that more stereotypic movements and less dystonic features occur in post-grafting rats might account for this deference in designing (34), as the latter was initially designed for dopaminergic neuron transplantation-related studies. In their attempt to distinguish between choreiform and dystonic movements, Steece-Collier's scale is similar to that used in the clinic and seems to be more accurate in recording of rodent dyskinesia, but a single-point scoring only at the peak-dose period was considered a limitation.

**Table 1:** Current options used to induce and evaluate LID in unilateral 6-OHDA-lesioned rat models.

	**Henry's protocol** **(Rotational sensitization)**	**Cenci's protocol** **(AIMs rating scale)**	**Steece-Collier's protocol**
**MODELING PROCEDURES**			
Lesion sites	MFB	MFB/striatum	MFB
Screening	Spontaneous rotation	Amphetamine-induced rotation	Amphetamine-induced rotation
LID inducing (drugs and dosage)	Methyl L-DOPA 6.5 mg/kg/day	Methyl L-DOPA 6 mg/kg/day	Methyl L-DOPA 50 mg/kg/day
	Benserazide 25 mg/kg/day	Benserazide 25 mg/kg/day	Carbidopa 5 mg/kg/day
**LID ASSESSMENT**			
Rating targets	Rotational turns	The presenting time of locomotive, axial, limb, and orolingual AIMs	The presenting time and the intensity of dyskinetic movements in different parts of the body
Patterns of movements scored	Contralateral rotation	Rotational locomotion, axial torsion, limb movements, and orolingual stereotypies	Clasping of forepaw, twisting of axial musculature, torsional movements beginning in the neck region, and twisting of limbs.
Monitoring styles	A consecutive 120-min monitoring	A disconnected 180-min monitoring with 9 turns of rating, and each rating session lasts 1 min	A single-point 2-min rating 30 min after the administration of L-DOPA
Rating styles	Automated rotometry	Human observation	Human observation
Pros	Time-effective an objective measuring with high inter-rater consistency	Good validation in pharmacological and biomarker's tests	The precise recording of dyskinetic movements
Cons	Not supported by pharmacological validation	Relatively poor inter-rater reliability	Lack of pharmacological validation
	The predictive value of rotational sensitization is controversial		Single-point sampling at the peak time cannot cover the whole time–action curve of an agent

### 6-OHDA Mouse Model

MPTP injection is the classic approach used to induce PD in mice. However, MPTP mice exhibit a prominent capacity for motor function recovery instead of persistent dysfunction; thus, they are suitable for molecular and cellular mechanisms studies rather than studying the symptomatology of PD (42). Indeed, some scholars have attempted to induce LID in MPTP mice with a high dose of L-DOPA and found that such dose causes few dyskinetic subtypes, unlike that observed in the non-human primate model or 6-OHDA-lesioned rat model (43). The study of LID in mice lagged far behind that in rats. It was the case until unilateral 6-OHDA-lesioned mice were introduced by Cenci's group and a similar AIM rating scale for mice was built up in 2004 (44). The advantage of the 6-OHDA lesion procedure lies in its stable and duplicable damage to the nigrostriatal DA neurons, besides its high predictability during the time course of DA degeneration (8). Compared with the rat, mouse AIMs are more rapid, have a more simplified repertoire, and present more prominent rotational locomotion with less dystonic features, i.e., axial AIMs (44, 45). High postoperative mortality used to be a major obstacle to the development of mouse models, especially those lesioned in the medial forebrain bundle (MFB), but recent progress in surgical procedures and postoperative support have improved this situation (46, 47).

## Validation of Rodent Models

Animal models play an irreplaceable role in the search of new medications for LID. It is important for a model to be validated. There are two main aspects of validity referred to here: predictive validity and construct validity. Predictive validity reflects how well the animal model imitates humans in response to experimental manipulation; thus, naturally it can be probed by testing the model's response to the effects of pharmacological agents known to alleviate or aggravate LID in the clinic. While construct validity examines whether the test performance is based on the actual neurobiological mechanisms underlying the disease. Accordingly, this is determined by quantifying dyskinesia-associated molecular markers in the animal model (48). Henry's group first compared the rotational behavior in rats caused by L-DOPA and bromocriptine (a DA agonist known to present low dyskinesiogenic potential when clinically used for PD treatment). They pronounced that there was no significant sensitization in the bromocriptine group (39). However, such a conclusion was met with strong criticisms (28, 49). Cenci's protocols have been tested by other agonists with low dyskinesiogenic potential including quinpirole, pramipexole, ropinirole, and bromocriptine, etc. in rats (13, 28, 50) and mice (45). These tests highlighted a good predictive validity of the rodent AIMs in line with the measures of dyskinesia that are used in both the clinic and non-human primate models. Moreover, L-DOPA-induced ALO AIMs could be ameliorated by clinically confirmed antidyskinetic compounds including amantadine, clozapine, and yohimbine in rats (28, 29), which further supports AIM scores (locomotive AIM excepted) as a reliable assessment tool for dyskinesia. Although few pharmacological validations for Steece-Collier's protocol have been reported, a good correlation has been recently demonstrated between this protocol and that developed by Cenci (41).

It was previously demonstrated that striatal mRNA's level of opioid precursors and related proteins, i.e., preproenkephalin-A (PPE-A), preproenkephalin-B (PPE-B), the 67-kDa isoform of glutamic acid decarboxylase (GAD67), closely, if not specifically, correlated with dyskinesia expression in primate PD models (7), which were also the postsynaptic markers of basal ganglia output pathways. To test the construct validity of Henry's protocol, several groups have reported upregulation of PPE-A, PPE-B (39), or their precursor Pdyn (24) mRNA level in response to rotational sensitization in rats treated repeatedly with L-DOPA; however, the lack of subgroup analysis in these studies made it difficult to confirm that the upregulation of these markers is a specific response to rotation behavior other than the general effects of drug treatment. In Cenci's protocol, better experimental controls were designed, which highlighted a linear positive correlation between ALO AIM scores and the expression level of these molecules in rats (25–27) and mice (44, 51). Also using Cenci's protocol, a highly interactive and positive correlation was found between the expression level of transcription factor FosB/ΔFosB and dyskinesia severity in rats (27) and mice (44), which was later confirmed to occur in PD patients (52, 53) and non-human primate models (54, 55). Overall, these studies demonstrate the validity of Cenci's protocol. A similar validation for Steece-Collier's protocol has also been done, but only in a few reports (34, 35).

## Controversies Around the Assessment of Rodent Dyskinesia

### The Predictive Value of the Rotation

Few behaviors are more impressive than drug-induced rotation in hemiparkinsonian rodents. DA agonists induce contralateral rotation, while agents that increase DA levels, i.e., amphetamine and amantadine, cause ipsilateral rotation. The physiological mechanism of this specific behavior has not been completely elucidated (28, 56). However, the dominant DA-dependent hypothesis advocates that circling is the result of a bilateral dopaminergic imbalance in the striatum and animals always tend to rotate toward the side with lower striatal dopaminergic activity (57).

The interpretation of rotation has always been the focus of debate. For a long time, the intensity of rotation was regarded to be a correlative index of the degree of neurotoxin-induced DA denervation for screening well-lesioned animals, as well as an indicator for evaluating the anti-akinetic efficiency of antiparkinsonian drugs (12, 58, 59). An inherent contradiction of this theory lies in the following reasoning: if the intensity of rotation is equivalent to the therapeutic effects, then the sensitization of rotational behavior should naturally be interpreted as an enhanced therapeutic action, which is apparently inconsistent with the clinical fact that the efficacy of L-DOPA always decreases gradually as the medication persists (12). Moreover, L-DOPA-induced rotation is a purposeless movement that is poorly correlated with the improvement of motor function as determined by cylinder test or rotarod test (28, 60), as sensitive measures of motor impairments in hemiparkinsonian rodents. Presently, majority of studies have reached a consensus that the measure of rotation should not be included in the AIM score (29, 49, 61, 62), and the shortened duration of rotation response should be viewed as a behavioral index of “wearing-off” like phenomenon (59, 61), but the behavioral significance of rotation requires further clarification.

### Environmental Interference

Environmental factors, mainly the test apparatus in which dyskinesia and rotation measures are performed, have not been considered as a source of interference. Various designs and sizes of test apparatus are employed in different laboratories, including hemispheric bowl (i.e., automated rotometry), rectangular boxes (i.e., rearing cages), and cylinder containers. Pinna et al. first reported that a more prominent sensitization of rotational behavior was induced when rats were tested in the hemispheric apparatus compared with rectangular boxes (63). However, this idea was challenged by other scholars (29, 41) as different counting procedures, i.e., automated rotometry and visual observation, were used in this case. Conversely, it was found that all AIM scores, Steece-Collier's scores, and the number of rotation turns in the Cenci model were not significantly different between round cylinders and square boxes (41).

Nevertheless, it should be noted that a number of previous studies that confirmed the environment-dependent behavioral response to DA agonists were performed in non-parkinsonian rodents (64–66). According to our observation (unpublished), motor response to L-DOPA is always disturbed or suspended when the rat moves to the corners of a rectangular box, and more dyskinetic features but less rotation behavior are presented in an enclosed and smaller container compared with the open field. A possible explanation for this difference might be that the container's walls facilitate a bipedal standing position in rats, which is associated with ALO AIMs (67). Based on these clues, it is reasonable to speculate that the test environment influences the rodent dyskinesia assessment; however, further supporting evidence is required to confirm this concept.

## Factors That Influence Dyskinesia Assessment in Rodents

### DA Denervation

Numerous studies have demonstrated a positive but non-linear correlation between AIM scores and the extent of DA denervation in rats (26, 27, 67) and mice (44, 45) by quantifying spared nigral DA neurons (determined by tyrosine hydroxylase immunohistochemistry) and/or striatal DA fibers (determined by DA transporter autoradiography). These studies found that a residual DA innervation below a critical, threshold value is a necessary prerequisite for animals to develop AIMs. In rats, it has been reported that <10% of DA cell sparing is needed for overt ALO AIMs, while a stricter <5% DA cell sparing is required for locomotive AIM (26). This is in line with the mainstream findings that DA denervation is a necessary but not sufficient condition for the development of dyskinesia in patients (68).

### Animal Individual Differences

Similar to human dyskinesia, hemiparkinsonian rodents exhibit significant differences in latency, severity, and subtypes of AIMs across individuals, although these features are relatively constant in one animal (27, 67). This cannot be only interpreted by the degree of DA denervation as AIM scores show a dispersed distribution even among rats with most severe denervation. Although the mechanisms are not fully understood, it has been proposed that the presence of non-dyskinetic animals and a high inter-individual variability in AIMs severity might contribute to understanding the factors that promote human dyskinesia (8, 69).

### 6-OHDA Lesion Sites

For nigrostriatal lesion, two main intracranial injection sites are used: MFB and striatum. The classical MFB model produces a fast (<3–4 days) and severe (usually <20% residual innervation) DA depletion that imitates the advanced stage of PD, while striatal lesion leads to a protracted and moderate retrograde degeneration (1–3 weeks) more similar to that seen in parkinsonian patients; for a review, see (42). A study by Winkler et al. compared dyskinetic features between these two models and found an overall lower incidence, less severity, and different topographic distribution of AIMs in the latter model under the same L-DOPA dosage (26). Importantly, there was no locomotive AIM (or rotation) in intrastriatally lesioned rats. This may be ascribed to the fact that locomotion in rodents is mediated by DA fibers in the medial subregion of striatum, and intrastriatal administration of 6-OHDA triggers a focal destruction only in the ventrolateral striatum, but not in the medial striatum. In contrast, the MFB lesion damages the DA fibers in the whole striatum (26, 27). Correspondingly, intrastriatally lesioned mice were less likely to develop dyskinesia compared to MFB-lesioned ones, but there was no difference in the representation of AIM subtypes (44). It was proposed that different brain sizes might account for this inter-species variation.

An unusual characteristic of striatum-lesioned mice is the decline of AIM severity after a few weeks of L-DOPA treatment (45). The mechanism for this observation is poorly understood, but a postsynaptic hypothesis, *viz*., the desensitization of postsynaptic D1 receptor (45), and the shortening of the motor response duration to L-DOPA (19, 45, 70) similar to the “wearing-off” phenomenon of one of the late complications of drug treatment for PD may account for this.

### The Dosage Regimen of L-DOPA and Benserazide

A dose-dependent relationship has been demonstrated between L-DOPA and the intensity of rotation, rotational sensitization, and AIM scores (49). Given its higher water solubility, L-DOPA methyl ester hydrochloride (methyl L-DOPA) is commonly used in many laboratories instead of ordinary L-DOPA. However, the dose of methyl L-DOPA adopted in previous studies varies from 2.5 to 50 mg/kg, making it challenging to directly compare results between such studies. Cenci and Lundblad proposed 6–10 mg/kg per injection as a standard dosage for rats and MFB-lesioned mice in dyskinesia studies, but a three- to four-fold dosage is required for intrastriatally lesioned mice to yield similar dyskinetic responses (71). It was reported that a standard dose regimen produced a pronounced interindividual variation of dyskinesia severities, but a high-dose regimen (usually >25 mg/kg/day) evoked a more uniform response with rotation in all animals (69). Besides the dosage per injection, administration frequency may also influence animal behaviors. Rodents receiving frequent injections maintained a hyperactive state for a longer time during a day, but application of the dose once or twice per day is recommended to be sufficient for dyskinesia studies (71).

Benserazide, a peripheral inhibitor of aromatic amino acid decarboxylase (AADC), is concomitantly used with L-DOPA to increase its effective concentration in CNS while reducing its peripheral side effects. It has been found that benserazide prolongs the acting time of L-DOPA dose-dependently in rodents (72). According to Cenci's report, when benserazide was applied at a dose of <8 mg/kg per day, the duration of L-DOPA action lasted for 100 min, which diminished the AIM scores. In contrast to the fixed ratio of L-DOPA:benserazide, 4:1 in standard clinical preparations, the dosage of benserazide for rodents varies greatly among studies; thus, a dose of 12 to 15 mg/kg/day has been proposed to be the appropriate regimen for inducing rodent dyskinesia (71).

### The Length and Turns of Monitoring

As mentioned above, a long enough monitoring is important for evaluating the anti-dyskinetic potential of different drugs, especially those with unknown pharmacodynamic profiles. The original version of Cenci's protocol adopted a 180-min monitoring with nine turns of scoring (25, 27, 44), but different lengths (such as 60, 120, 160, and 240 min) and turns (2, 4, 8, and 12, respectively) of monitoring have also been used currently (31, 32, 73, 74). Whatever length is employed, it must be able to cover the whole time–action curve of the agents being investigated. Furthermore, a multiple time-point evaluation has been found to be superior to selected peak-time assessment adopted in Steece-Collier's protocol, as some agents, e.g., amantadine, may reduce dyskinesia severity at peak time, but prolong the duration of motor dysfunction (41).

### Priming Effect

Priming refers to the phenomenon in which a single or repeated exposure to specific DA agonists would lead to a long-lasting enhancement of locomotor and stereotypic behaviors elicited by the subsequent DA agonist challenge (75). A study showed that twice administration of apomorphine 1 month earlier would remarkably promote rotation behavior and AIMs during the subsequent chronic DA replacement therapy, inducing not only a higher degree of turning and AIM scores but also an earlier emergence of their peak value, which occurred on the first rating day (13). Thus, the priming effect should be seriously taken into consideration, as dopaminergic drugs, e.g., apomorphine, is commonly used for screening well-lesioned animals. Alternative drug-free tests such as the cylinder test have been found to be effective in avoiding such interference (71).

### Strain Differences

Unlike MPTP, the sensitivity to 6-OHDA among different strains of rats and mice are relatively stable (42); thus, strain differences are always not considered in rodent dyskinesia studies. So far, almost all published studies were carried out in Sprague Dawley rats, Wistar rats, or C57BL6 mice. Thiele et al. compared the rotation behavior and AIMs of pure FVB and FVB/C57BL6 mice following a chronic L-DOPA treatment. They observed that the latter were more sensitive to develop dyskinetic behaviors under the same dosing regimen (76). No other strain comparisons have been reported so far, but such a latent interstrain difference should be considered as different strains of transgenic mice might be used for dyskinesia studies in the future.

## Current Usage and Advances in the Use of Rodents in Dyskinesia Studies

Given the developments witnessed in the past two decades, the use of rodents as useful models for dyskinesia has gradually become mainstream in academia (8, 69). Cenci's protocol has become the most widely accepted option for evaluating dyskinesia in 6-OHDA-lesioned rodents. On the basis of this protocol, numerous pharmacological trials have led to the discoveries of several promising therapeutic targets in recent years, including the GABA system (77–79), the serotonin system (80–84), adenosine receptors (85–88), opioid receptors (89–92), neuronal nitric oxide synthase inhibition (93), β_2_ nicotinic receptors (94–98), and cation channels (99, 100). Confidence in the application of rodent models has been further boosted by the findings that the GABA system and serotonin system translate quite well when these agents are applied in non-human primate models and clinical trials (8). Otherwise, due to the ease of genetic engineering (both knock-in and knock-outs), the mouse has become the standard model for studying molecular pathways of dyskinesia. This model has led to the discovery that the ERK1/2-DARPP32-mTOR and Ras-GRF1 signal pathways play important roles in the development of LID (51, 55, 101–103). Previous studies have shown that changes in synaptic plasticity influence the pathogenesis of dyskinesia (104, 105). It has been demonstrated that bidirectional synaptic plasticity exists in the cortical striatum of normal rats, including long-term potentiation (LTP) and long-term depression (LTD). This bidirectional synaptic plasticity is essential in maintaining stable motor function (106). For LID rats, the indirect pathway is only characterized by LTD, while the direct pathway is characterized by LTP (107), which leads to the clinical manifestation of dyskinesia. In addition, many therapeutic approaches targeted at pathways such as mTOR pathway (108, 109), Ras-ERK signaling (110, 111), M4 muscarinic receptor signaling (107), and metabotropic glutamate receptors (112, 113) have shown high potential in alleviating LID in rats. In recent years, multi-channel electrophysiological recording technology has enabled synchronous observation and recording of LFP, spike signals, and behavior in multiple brain regions, which paves the way for a deeper understanding of the mechanism of abnormal neural loops in PD and dyskinesia. The β-oscillation associated with motor inhibition in the cortical–basal ganglia circuit (114, 115) and the gamma oscillation associated with dyskinesia (116, 117) have been confirmed in various animal models such as rats, mice, non-human primates, and human experiments. Although the mechanisms of the electrophysiological abnormalities are not fully known, the relationship between electrophysiological indicators and pathological status provides new avenues for evaluating the efficacy of drugs on dyskinesia.

Over the past 10 years, a number of discoveries have been made, which have expanded our understanding into the role of rodent models in LID studies. Paille et al. established the first bilateral 6-OHDA-lesioned rat model for dyskinesia study, although this model was characterized by unilateral AIMs (118). Interestingly, a study reported that asymmetric severity of AIMs was induced in the left, right, and inverse sequential bilateral lesioned rats (119). This may have a profound effect on rodent LID models if it is proven to be correct in further studies. Although tremendous progress has been made in developing genetic animal models of PD in recent years, there are still many challenges affecting behavioral research. One of the key limitations is that these models cannot reproduce the degeneration of dopaminergic neurons observed in PD patients. For mouse models used to study gene overexpression involving some familial PD forms, such as α-synuclein and leucine-rich repeat kinase 2, since they do not experience dopaminergic cell loss, LID cannot be induced with levodopa treatment (120), and there is currently no single PD animal model that perfectly replicates all the core features of PD (121). Nevertheless, this model still plays an important role in exploring the molecular mechanisms of early pathological changes in PD. It has been reported that RGS9 knockout mice are induced to dyskinesia when subsequently activating D2-like dopamine receptors (DRs) following inhibition of dopaminergic transmission (122). Pitx3^ak/ak^ mice, or aphakia mice, are established by deletion of the Pitx3 promoter region. Aphakia mice display a spontaneous recessive phenotype characterized by lack of lens in the small eyes (123) and during development. In this mice, both sides of the cerebral hemisphere lack the nigrostriatal DA projection and hence are more likely to develop LID. In 6-OHDA and other drug-induced PD mouse models, it is not easy to achieve bilateral DA depletion without excessive mortality (124). Cao et al. (125) injected a virus vector carrying ΔFosB cDNA into the injured striatum of rats, and Feyder et al. (126) developed a PD model of mitogen and stress-activated kinase 1 knockout mice (MSK1KO) and ΔFosB- or ΔcJun-overexpressing transgenic mice with 6-OHDA. In both studies, chronic L-DOPA treatment successfully induced LID.

## Conclusions

The development of unilateral 6-OHDA-lesioned rodent models and the establishment of corresponding behavioral assessment protocols have greatly promoted the research of dyskinesia over the past two decades. This has led to the discovery of several novel therapeutic agents to control the intractable complications advanced PD. In spite of the controversies surrounding the behavioral assessment in these models, rodent models are still powerful and cost-effective tools in dyskinesia studies. In order to properly assess abnormal involuntary movement of PD rodents and to accurately conduct related mechanisms and drug development studies, these behavioral assessment methods and rating scales need to be used rationally.

## Author Contributions

QP, SZ, YT, WZ, JW, CC, YW, and XY reviewed the literature and drafted the manuscript. YX and XC revised and proofread the manuscript. All authors read and approved the final manuscript.

### Conflict of Interest

The authors declare that the research was conducted in the absence of any commercial or financial relationships that could be construed as a potential conflict of interest.

## Abbreviations

PD: Parkinson's disease
DA: dopamine
L-DOPA: levodopa
LID: L-DOPA-induced dyskinesia
GID: graft-induced dyskinesia
MPTP, 1-methyl-4-phenyl-1, 2, 3: 6-tetrahydropyridine
6-OHDA: 6-hydroxydopamine
AIM: abnormal involuntary movements
MFB: medial forebrain bundle
PPE-A: preproenkephalin-A
PPE-B: preproenkephalin-B
GAD: glutamic acid decarboxylase
AADC: aromatic amino acid decarboxylase
CNS: central nervous system
GABA: γ-aminobutyric acid
ERK1/2: extracellular regulated kinase 1 and 2
DARPP-32: phosphoprotein of 32 kDa
m-TOR: mammalian target of rapamycin
GRF1: guanine nucleotide exchange.
